# Multiregional profiling reveals *THBS1*-*SPP1* monocyte-macrophage axis drives immunosuppression and outcome in colorectal liver metastases

**DOI:** 10.1126/sciadv.aed1296

**Published:** 2026-05-22

**Authors:** Gaia Bellomo, Jayden Gittens, Christopher Brunning, Maidinaimu Abudula, Robert P. Jones, Michael C. Schmid, Ainhoa Mielgo

**Affiliations:** Department of Molecular and Clinical Cancer Medicine, University of Liverpool, Ashton Street, Liverpool L69 3GE, UK.

## Abstract

Colorectal cancer (CRC) commonly metastasizes to the liver (CRLM), where it is the leading cause of CRC-related deaths. While immune checkpoint therapies show promise, their effectiveness is limited in CRLM due to the immunosuppressive liver tumor microenvironment (TME). Using multiregional tissue sampling from CRLM patient samples, we identified distinct immune zones within CRLM. Active Granzyme^+^CD8^+^ T cells were found in normal liver tissue, while CD8^+^ T cells in the tumor core were dysfunctional. At the tumor margin, we observed a pre-exhausted immune zone enriched in *THBS1^+^* monocytes and CD47*^+^*CD8*^+^* T cells. Trajectory analysis showed that *THBS1^+^* monocytes differentiate into *SPP1^+^* macrophages, which accumulate in the tumor core and promote immune suppression via *TIM-3* and *CTLA-4* on exhausted CD8^+^ T cells. The presence of *SPP1^+^* macrophages correlates with increased T cell exhaustion and poor survival, suggesting them as potential targets to restore antitumor immunity in CRLM.

## Teaser

Immune profiling of CRLM identifies *SPP1*-macrophages as key mediators of T cell exhaustion and potential targets for therapeutics.

## INTRODUCTION

Metastasis is the leading cause of cancer-related mortality (*1*). In colorectal cancer (CRC), the 5-year survival rate for patients with metastatic disease is only 12%, largely driven by liver involvement. Approximately half of the patients with CRC develop detectable liver metastases within 5 years of their primary tumor diagnosis (*2*, *3*). Although surgical resection combined with perioperative chemotherapy offers curative intent for colorectal liver metastasis (CRLM), up to 65% of patients experience disease recurrence within 5 years, underscoring the urgent need for novel therapeutic strategies targeting metastatic disease (*4*).

The CRLM tumor microenvironment (TME) is a complex and dynamic ecosystem. Immune cells constitute the most abundant noncancerous population (*5*–*7*), with their function shaped by local environmental cues and activation states. Immune infiltration and activity vary spatially within the TME, where tumor-promoting and tumor-suppressing cells coexist (*8*, *9*). The tumor core is largely immunosuppressed, whereas circulating immune cells often display an immune-stimulatory phenotype that diminishes upon entering the tumor (*5*, *6*, *10*). Our understanding of the mechanisms and the timing of immune inactivation has been limited by the lack of single-cell resolution and restricted spatial coverage in previous transcriptomic studies (*11*).

To address this, we applied single-cell RNA sequencing (scRNA-seq) to multiregional fresh CRLM tissue samples, revealing distinct spatial immune compartmentalization. We identified three key zones: (i) immune-active regions in noninvolved liver (NIL) tissue enriched with active CD8^+^ T cells; (ii) a pre-exhaustion zone at the tumor edge characterized by interactions between *THBS1^+^* monocytes and *CD47^+^CD8^+^* T cells; and (iii) an immune-silenced tumor core enriched in exhausted *TIM-3^+^CTLA4^+^* T cells. *THBS1^+^* monocytes may differentiate into *SPP1^+^* macrophages within the tumor core, which perpetuate immunosuppression through checkpoint engagement on exhausted T cells. Thus, our findings reveal that targeting *SPP1^+^* macrophages and *TIM-3/CTLA4* checkpoints may represent promising strategies to sustain CD8^+^ T cell activation and cytotoxicity in CRLM.

## RESULTS

### Multiregional scRNA-seq reveals spatially structured immune zones in CRLMs

**Fig. 1.**
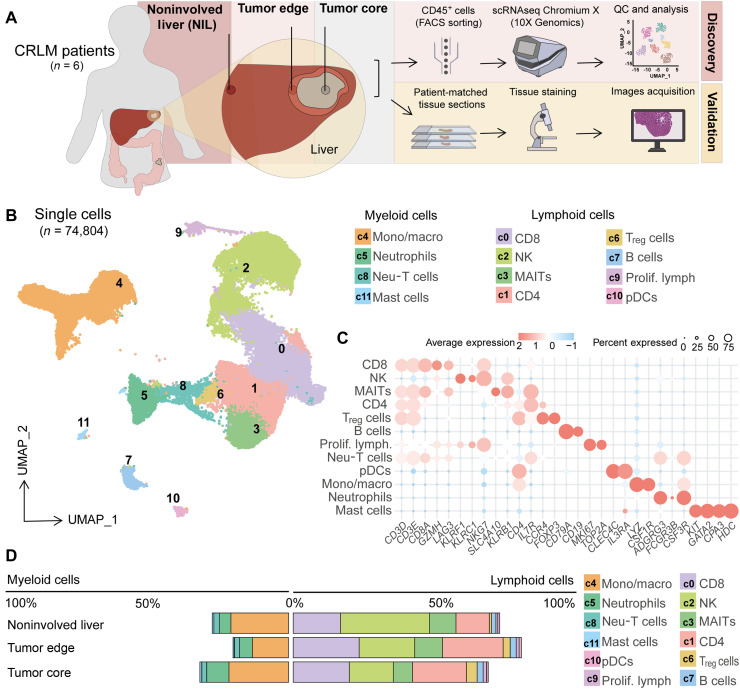
scRNA-seq reveals distinct immune cell populations within different regions of CRLM. (**A**) Schematic depicting the sampling of CRLMs from the NIL, tumor edge, and tumor core, followed by tissue processing, CD45^+^ immune cell sorting, scRNA-seq, and data analysis. Complemented by patient-matched microscopy-based tissue analysis. (**B**) Uniform Manifold Approximation and Projection (UMAP) plot identifying 12 clusters within CD45^+^ cells. (**C**) Dot plot displaying lineage gene markers used for cluster annotation. (**D**) Stacked histogram showing the proportion of major immune cell populations across CRLM regions, split by lineage: myeloid (left) and lymphoid (right). pDCs, plasmacytoid dendritic cells.

To unravel the intricate immune landscape within CRLM, we conducted scRNA-seq on CD45^+^ isolated cells from resected liver specimens of six clinically annotated patients with CRLM (table S1). We collected matched samples from three spatially distinct regions: the tumor core, tumor edge, and adjacent NIL tissue (Fig. 1A and figs. S1A and S2A).

After quality control and normalization, our dataset comprised 74,804 cells (fig. S2, B and C). Integrating data across all patients and anatomical sites, we applied unbiased clustering, revealing a rich diversity of immune cell populations (Fig. 1, B and C, and fig. S2, D to F). Using differentially expressed genes (DEGs) and lineage-defining markers, we confidently annotated major cell types, including cytotoxic CD8^+^ T cells, helper CD4^+^ T cells, natural killer (NK) cells, mucosal-associated invariant T (MAIT) cells, monocytes/macrophages, neutrophils, regulatory T cells (T_reg_ cells), B cells, proliferative lymphoid cells, plasmacytoid dendritic cells, and mast cells (table S2). One unusual population expressing mixed lymphoid and myeloid markers was excluded from further analysis (Fig. 1, B and C).

Examining overall abundance, NK cells emerged as the most prevalent immune population (22.7%), followed closely by CD8^+^ T cells (19.3%), monocytes/macrophages (17.9%), and CD4^+^ T cells (16.6%). MAIT cells and neutrophils also formed substantial fractions, while T_reg_ cells, B cells, and others appeared less frequently (fig. S2F). These findings align well with prior studies (*5*, *6*) and were validated by mass cytometry on additional CRLM samples (table S1 and fig. S3, A to C).

Notably, spatial analysis uncovered profound differences in immune cell distribution across tumor regions. Myeloid cells were enriched in both NIL and tumor core but particularly sparse at the tumor edge. NK cells predominated in NIL but steadily declined toward the tumor core. In contrast, the tumor edge and core harbored increased numbers of CD4^+^ T cells and T_reg_ cells (Fig. 1D). Collectively, these data reveal a highly compartmentalized immune architecture in CRLM, with distinct immune “zones” shaping the TME.

### Spatial and functional diversity of T cell subsets shapes the immune landscape in CRLMs

CD4^+^ and CD8^+^ T cells are abundant and phenotypically diverse across CRLM immune zones. Unsupervised clustering identified 11 CD4^+^ subsets, including T helper 17 (T_H_17), T follicular helper (Tfh), cytotoxic CD4^+^ effectors (ThCTL), and T_reg_ cells (fig. S4, A and B, and table S3). Spatially, ThCTLs were enriched in NIL but decreased toward the tumor core; T_H_17 cells clustered at the tumor edge, while Tfh and T_reg_ cells increased from edge to core (fig. S4, C and D).

**Fig. 2.**
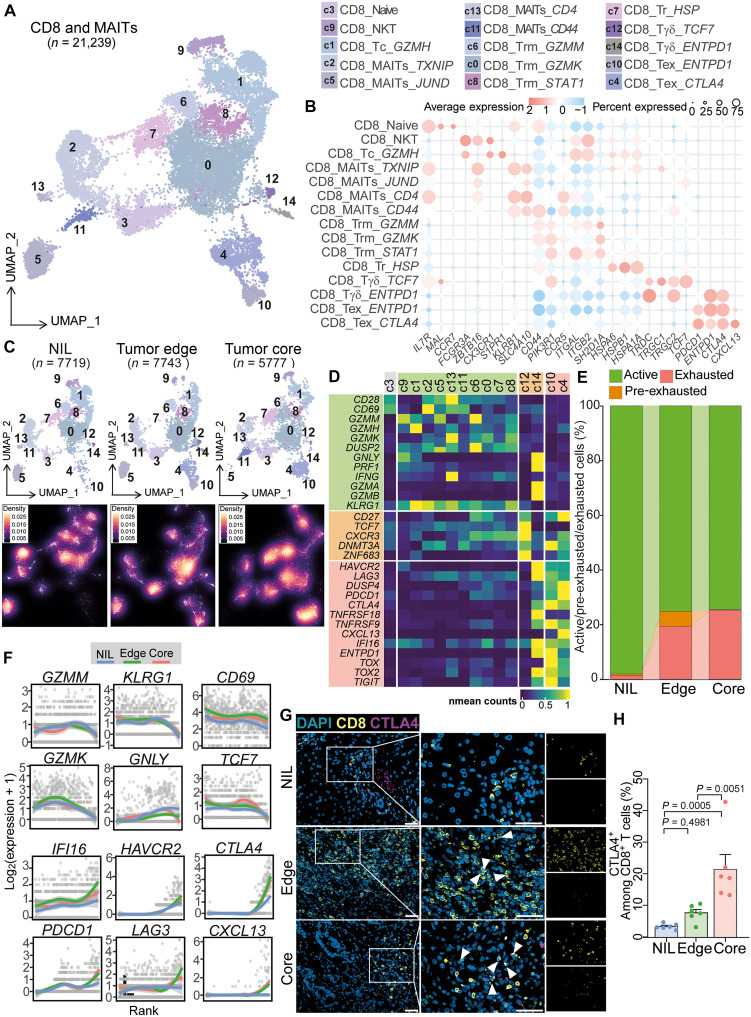
Progressive exhaustion of CD8^+^ T cells from NIL region to tumor core. (**A**) UMAP plot identifying fifteen CD8^+^ T cell clusters. (**B**) Dot plot displaying gene markers used for cluster annotation. (**C**) UMAP view of CD8^+^ T cell clusters (top) and cell density (bottom) displaying CD8^+^ T cell distribution across NIL, tumor edge, and core. (**D**) Heatmap showing normalized average expression of selected genes associated with T cell activation and/or exhaustion for each CD8^+^ T cell cluster. (**E**) Stacked bar plot depicting the proportion of active, pre-exhausted, and exhausted CD8^+^ T cells [cell state defined in (D)], across the NIL, tumor edge, and tumor core. (**F**) Single-cell expression of the indicated cytotoxic and exhaustion genes ordered by pseudotime. The lines correspond to locally estimated scatterplot smoothing (LOESS) curve for each zone (NIL, tumor edge, and tumor core). (**G**) Representative immunofluorescent images of exhausted CD8^+^ T cells (CD8^+^CTLA4^+^) in NIL, tumor edge, and tumor core. Scale bars, 50 μm. (**H**) Quantification of exhausted CD8^+^ T cells (CD8^+^CTLA4^+^) in NIL, tumor edge and tumor core.

For CD8^+^ T cells, 15 distinct clusters were found, covering naïve-like, circulatory, NKT, MAIT, γδ, tissue-resident memory (Trm), and exhausted populations (Fig. 2, A and B). Trm cells expressed residency markers (*CD44* and *CCR5*), while exhausted cells showed high immune checkpoint expression (*PDCD1* and *CTLA4*) (table S4). Spatially, circulatory CD8^+^ T cells dominated NIL; γδ and exhausted *ENTPD1^+^* cells were enriched at the tumor edge, and heat shock protein-expressing plus terminally exhausted CD8^+^ T cells were most abundant in the tumor core (Fig. 2C and fig. S4, E and F). This reveals clear spatial specialization of T cell subsets in CRLM.

### Multiregional profiling of functional CD8^+^ T cell states reveals the tumor edge as a transition zone toward exhaustion

To characterize the functional diversity of CD8^+^ T cells in CRLM, we classified cells based on activation and exhaustion gene signatures. Resting cells (cluster c3) lacked cytotoxic or inhibitory features, while activated populations expressed granzymes (*GZMA* and *GZMK*), perforin (*PRF1*), *IFNG*, and surface markers such as *CD28* and *CD69*. Notably, two clusters (c12 and c14) exhibited a pre-exhausted phenotype, defined by the coexpression of activation and inhibitory genes or by the expression of transitional markers such as *TCF7* and *CXCR3*, indicative of a state poised between effector function and exhaustion (*12*, *13*). Terminally exhausted clusters (c4 and c10) were marked by high expression of *PDCD1*, *CTLA4*, *LAG3*, and *HAVCR2* (Fig. 2D).

The spatial mapping of these states revealed a clear zonation: Activated CD8^+^ T cells were enriched in the NIL, pre-exhausted cells localized predominantly at the tumor edge, and terminally exhausted cells accumulated within the tumor core (Fig. 2E). This spatial organization was further supported by examining CD8^+^ T cell state-associated gene expression across tissue regions. Genes associated with CD8^+^ T cell activation were predominantly expressed in the NIL, and there was a mixed expression of activation and exhaustion markers in the tumor edge. In contrast, exhaustion-associated genes were primarily expressed in the tumor core (fig. S5A). Together, these findings establish the tumor edge as a distinct immunological niche where CD8^+^ T cells begin to lose effector function, suggesting a key transition zone that may be therapeutically targetable.

### Pseudotime trajectories of CD8^+^ T cells reveal progressive exhaustion toward the tumor core in CRLM

To investigate whether CD8^+^ T cells undergo a stepwise transition from activation to exhaustion across CRLM regions, we applied the pseudotime inference tool *Ouija* to single-cell transcriptomic data. Ordering cells by expression of activation and exhaustion markers revealed a continuous trajectory: Early pseudotime was associated with activated markers (*CD69*, *GZMK*, and *TCF7*), predominantly in NIL-derived cells, while late pseudotime corresponded with exhaustion markers (*PDCD1*, *CTLA4*, *CXCL13*, and *HAVCR2*), enriched in tumor edge and core samples (Fig. 2F and fig. S5, B and C).

These transcriptomic findings were supported by spatial immunofluorescence, which showed CD8^+^CD69^+^ T cells localized mainly in the NIL (fig. S5, D and E), and exhausted CD8^+^CTLA4^+^ T cells concentrated at the tumor edge and core (Fig. 2, G and H). Mass cytometry further confirmed this gradient, with significantly higher levels of exhaustion markers T cell immunoreceptor with Ig and ITIM domains (TIGIT) and programmed cell death protein 1 (PD-1) in CD8^+^ T cells from the tumor core compared to NIL and edge regions (fig. S5, F and G).

Together, these data reveal that while active cytotoxic CD8^+^ T cells are initially recruited to metastatic liver lesions, their functionality progressively declines as they move inward. The tumor edge emerges as a key “pre-exhaustion” checkpoint, where some CD8^+^ T cells coexpress activation (*GNLY*, *PRF1*, and *GZMA*) and inhibitory (*HAVCR2*, *LAG3*, and *TNFRSF18*) markers, suggesting that these transitional cells may still be amenable to therapeutic rescue (*14*). This presents a strategic window to halt or reverse exhaustion and restore antitumor immunity.

### Spatially distinct *MARCO*^+^, *SPP1*^+^, and *TREM2*^+^ macrophage niches shape a tumor permissive microenvironment in CRLMs

**Fig. 3.**
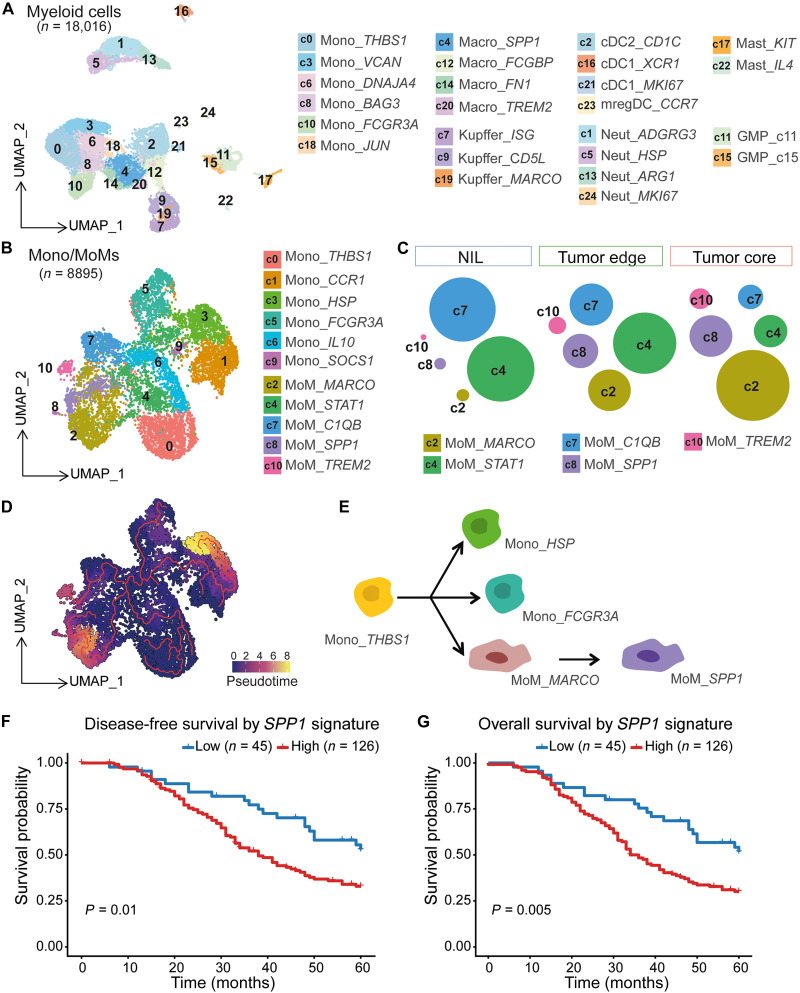
THBS1^+^ monocytes give rise to SPP1^+^ macrophages that localize to the tumor core and predict poor outcome. (**A**) UMAP plot identifying 25 myeloid clusters. (**B**) UMAP plot identifying 11 monocyte-derived clusters. (**C**) Diagram showing distribution of MoM clusters across NIL, tumor edge, and core. (**D**) UMAP plot illustrating single-cell transcriptomic data with an overlaid trajectory inferred using Monocle3. (**E**) Schematic representation of Monocle3-inferred differentiation trajectory from Mono_*THBS1* monocytes into distinct subsets, including Mono_*HSP*, Mono_*FCGR3A*, MoM_*MARCO*, and MoM_*SPP1*. Arrows indicate the direction of pseudotime progression. (**F**) Kaplan-Meier survival analyses of disease-free survival and (**G**) overall survival according to SPP1 signature (low versus high, defined by optimal cut point). Patients with high SPP1 signature had significantly shorter disease-free survival (DFS) (*P* = 0.01) and overall survival (OS) (*P* = 0.005).

To investigate how the myeloid compartment adapts to the CRLM microenvironment, we performed unsupervised clustering of CD45^+^ cells across the NIL, tumor edge, and tumor core. This, based on the top 50 principal components, revealed 25 distinct clusters (Fig. 3A). Using DEGs (table S5) and canonical myeloid markers (fig. S6A), we identified key monocyte and macrophage populations with region-specific abundance patterns. Monocytes dominated the NIL and tumor edge, while macrophages were strongly enriched in the tumor core (fig. S6B).

To dissect this further, we reclustered monocyte/macrophage populations, revealing 11 transcriptionally distinct subtypes all expressing core monocyte markers such as *CSF1R*, *CD14*, and *LST1* (Fig. 3B and fig. S7A). Five clusters coexpressed macrophage-associated genes (*CD68*, *MRC1*, *FN1*, and *APOE*), consistent with monocyte-derived macrophage (MoM) identities (c2, c4, c7, c8, and c10) (fig. S7B and table S6).

Spatial mapping uncovered highly dynamic abundances particularly within the MoM clusters between the three locations. Cluster c7 (*C1QB^+^*) was enriched in NIL, suggesting a role in homeostatic immune surveillance. In contrast, *SPP1^+^* (c8) and *TREM2^+^* (c10) macrophages were enriched at both the tumor edge and core, while *MARCO^+^* macrophages (c2) were found almost exclusively in the tumor core (Fig. 3C and fig. S7, C to E). The functional analysis of these clusters revealed specialized roles in shaping the immunosuppressive niche: *SPP1^+^* macrophages (c8) expressed genes linked to extracellular matrix remodeling and T cell suppression. *TREM2^+^* macrophages (c10) showed a lipid metabolism signature, often associated with tumor-promoting activity. *MARCO^+^* macrophages (c2) expressed chemotactic factors that may support continuous monocyte recruitment to metastatic sites (fig. S7F and table S6).

Together, these findings uncover a coordinated network of immunosuppressive macrophage subsets that occupy distinct spatial niches in CRLM. By performing complementary functions—ranging from immune evasion to structural remodeling—*SPP1^+^*, *TREM2^+^*, and *MARCO^+^* macrophages collectively contribute to the establishment and maintenance of a tumor-permissive microenvironment.

### Differentiation of *THBS1^+^* monocytes into *SPP1^+^* macrophages at the tumor edge promotes immunosuppression in CRLMs

Among the monocyte/macrophage clusters, the largest and most striking population consisted of *THBS1^+^* monocytes (Mono_c0_*THBS1*). Spatial mapping revealed that these cells are enriched in the NIL and tumor edge but become scarce within the tumor core, suggesting a transitional role at the tumor margin (fig. S7, C to E).

We hypothesized that *THBS1^+^* monocytes act as precursors to immunosuppressive macrophages (MoMs) within the CRLM TME. To test this, we performed trajectory inference using Monocle3, which revealed a clear differentiation path from Mono_c0_*THBS1* to downstream MoM subsets, including Macro_c2_*MARCO* and ultimately Macro_c8_*SPP1* (Fig. 3, D and E). This supports previous reports that *THBS1*-expressing monocytes give rise to *SPP1^+^* macrophages at primary tumor sites (*15*).

Clusters along this trajectory retained enriched angiogenesis-related gene signatures, indicating that monocytes and their macrophage progeny may sustain protumoral functions throughout their differentiation (fig. S7G and table S6) (*16*).

Tumor-associated macrophages have been found to correlate with poor prognosis (*17*–*19*). To assess the clinical relevance of the *SPP1^+^* population, we identified an *SPP1* macrophage gene signature derived from our scRNA-seq dataset, capturing the distinct transcriptional profile of *SPP1^+^* macrophages localized to the tumor edge and core (table S7). We then applied this signature to a publicly available CRLM microarray dataset to evaluate its association with clinical outcomes (*20*). The high expression of the *SPP1* macrophage signature was strongly associated with poor prognosis. Patients with elevated signature scores had significantly shorter disease-free and overall survival compared to those with lower expression (Fig. 3, F and G). These results highlight the potential of *SPP1^+^* macrophages as both a prognostic biomarker and a therapeutic target in CRLM.

Together, these findings reveal a conserved monocyte-to-macrophage differentiation trajectory in the metastatic liver, whereby *THBS1^+^* monocytes give rise to immunosuppressive *SPP1^+^* macrophages that drive tumor progression and worsen patient outcomes. Targeting this pathway may offer a promising strategy to disrupt the formation of an immunosuppressive niche in CRLM.

### Proximity to the tumor core rewires myeloid–T cell communication, driving T cell exhaustion in CRLMs

**Fig. 4.**
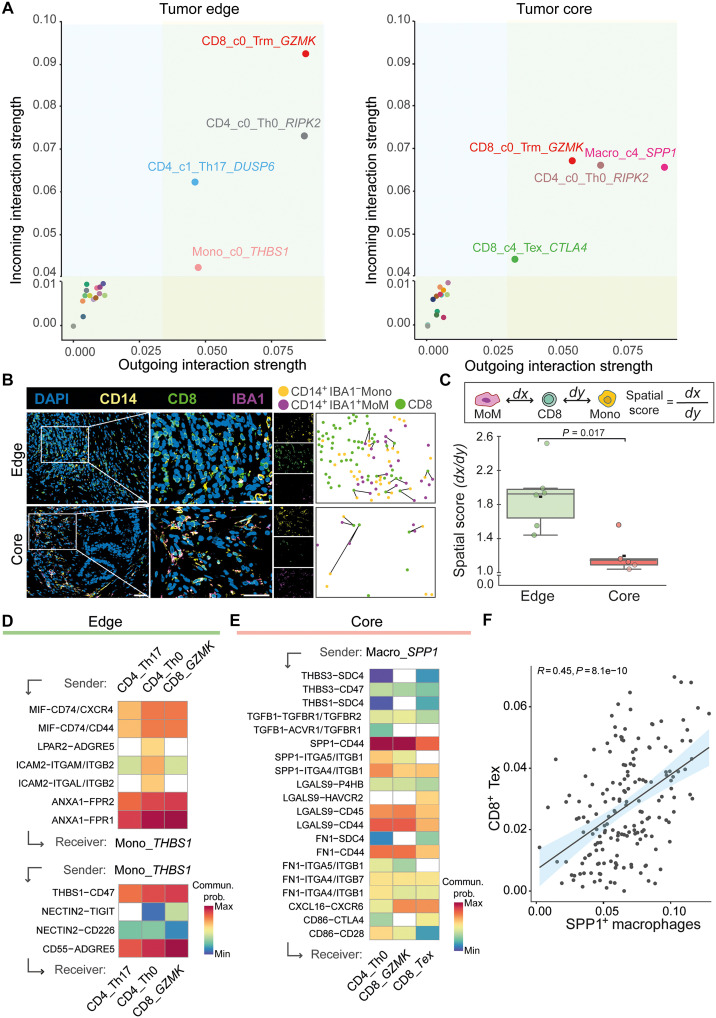
Monocyte/macrophage–T cell interactions are spatially regulated by proximity to the tumor core. (**A**) Graphs depicting main cluster interactors based on incoming and outgoing interaction strengths in the tumor edge (left) and in the tumor core (right). (**B**) Representative immunofluorescent staining (left) and corresponding spatial plots (right) of CD8^+^ T cells (CD8^+^), monocytes (Mono, CD14^+^IBA1^−^), and MoMs (CD14^+^IBA1^+^) in the tumor edge and tumor core. Scale bars, 50 μm. (**C**) SpatialScore calculated from immunofluorescent data per patient in tumor edge and tumor core. Error bars denotes means ± SEM. *P* values, Wilcoxon rank-sum test. (**D**) Heatmap depicting selected ligand-receptor interactions from T cell clusters to *THBS1^+^* monocytes (top) and LR interactions from *THBS1^+^* monocytes to T cell clusters (bottom), enriched in the tumor edge. (**E**) Heatmap depicting selected LR interactions from *THBS1^+^* monocytes to T cell clusters, enriched in the tumor edge. (**F**) Scatter plot shows the correlation between the abundance of *SPP1^+^* macrophages and exhausted CD8^+^ T (CD8^+^ Tex) cells in the CRLM microarray dataset GSE159216 (*n* = 171). The error band indicates the 95% confidence interval. In (D) and (E), color intensity represents the probability of communication. DAPI, 4′,6-diamidino-2-phenylindole.

To understand how spatial proximity to the tumor core shapes immune cross-talk in CRLM, we analyzed ligand-receptor interactions between CD4^+^ and CD8^+^ T cells and myeloid subsets using CellChat. This revealed notable regional differences in immune communication: At the tumor edge, *THBS1^+^* monocytes (Mono_c0_*THBS1*), CD8^+^ tissue-resident memory T cells (CD8_c0_Trm_*GZMK*) and CD4^+^ T_H_17 and T_H_0 cells formed a highly interactive network. In contrast, within the tumor core, immunosuppressive *SPP1^+^* macrophages (Macro_c4_*SPP1*) emerged as the dominant signaling hub, engaging primarily with exhausted CD8^+^ (CD8_c4_Tex_*CTLA4*) and CD4^+^ T cell subsets (Fig. 4A and table S8). To validate these predicted interactions, we performed multiplex immunostaining and applied the SpatialScore function to quantify the physical proximity of monocytes (CD14^+^IBA1^−^), macrophages (CD14^+^IBA1^+^), and CD8^+^ T cells (*21*). Consistent with CellChat findings, CD8^+^ T cells were significantly closer to monocytes at the tumor edge, whereas in the tumor core, they were more closely associated with macrophages (Fig. 4, B and C), indicating a spatial shift in immune regulation. Functionally, CD8^+^ Trm cells at the edge engaged *THBS1^+^* monocytes via *MIF-CD74/CXCR4* and *ANXA1-FPR1/2* signaling—pathways known to recruit and polarize monocytes—while CD4^+^ T_H_ subsets used similar axes (Fig. 4D, top, and fig. S8, A and B) (*22*, *23*). In turn, *THBS1^+^* monocytes were predicted to signal back to T cells via *THBS1-CD47*, which suppresses T cell receptor (TCR) signaling (*24*), and *NECTIN2-TIGIT*, promoting exhaustion (Fig. 4D, bottom, and fig. S8, C and D) (*25*). In the tumor core, *SPP1^+^* macrophages interacted with CD4^+^ T cells via *TGFB1-TGFBR1/2* to induce T_reg_ cell differentiation (*26*–*28*) and expressed *CXCL16*, *FN1*, and *SPP1*, which bind to *CXCR6*, integrins, and *CD44* on CD8^+^ T cells, facilitating infiltration (*29*), retention, and functional exhaustion (*30*–*32*) (Fig. 4E). These macrophages also expressed multiple inhibitory ligands (*LGALS9-HAVCR2* and *CD86-CTLA4*), reinforcing T cell dysfunction (fig. S8, E and F). Last, CIBERSORTx deconvolution of a public CRLM dataset (GSE159216) confirmed that *SPP1^+^* macrophage abundance positively correlated with exhausted CD8^+^ T cell levels (Fig. 4F). Together, these findings support a model in which immune cell positioning relative to the tumor core governs the nature of myeloid–T cell interactions, with *THBS1^+^* monocytes coordinating active immune cross-talk at the edge and *SPP1^+^* macrophages driving suppression in the core. This spatial reprogramming of immune communication may contribute to CD8^+^ T cell exhaustion and suggests that disrupting region-specific myeloid–T cell circuits could represent a therapeutic opportunity in CRLM.

## DISCUSSION

Using multiregional scRNA-seq, we mapped the immune landscape of CRLM and uncovered notable transcriptional diversity across three distinct zones: the metastatic tumor core, the tumor edge, and the distal NIL. Our analysis demonstrates that spatial proximity to the tumor core shapes immune cell localization, activation, and function, affecting both innate and adaptive compartments.

Consistent with previous studies, we found that CD8^+^ T cells in the NIL are transcriptionally active, while those infiltrating the tumor core express canonical exhaustion markers such as *CTLA4*, *HAVCR2*, and *TIGIT* (*5*). Crucially, the tumor edge emerged as a functionally distinct pre-exhaustion zone, where two subpopulations of CD8^+^ T cells—one expressing *TCF7* and another coexpressing activation and exhaustion markers—suggest a transitional phenotype amenable to therapeutic reactivation (*13*, *33*).

We also identified *THBS1^+^* monocytes at the tumor edge as key mediators of immunoregulation. These monocytes engage with *CD8^+^ GZMK^+^* Trm cells via the *THBS1-CD47* signaling axis, known to inhibit TCR-mediated activation (*24*). This interaction likely contributes to the early stages of T cell exhaustion. *THBS1* encodes thrombospondin 1, a matricellular protein that suppresses angiogenesis and activates transforming growth factor–β, linking *THBS1* to fibrosis and immune suppression (*34*, *35*). Supporting its clinical relevance, *THBS1* has been implicated in promoting metastasis in orthotopic CRC models, where *THBS1* knockout reduced liver and lymph node metastases in a CD8^+^ T cell–dependent manner. In addition, elevated serum thrombospondin 1 levels were observed in patients with CRC compared to those with benign lesions, highlighting its potential as both a biomarker and therapeutic target in metastatic CRC (*36*).

Recent large-scale myeloid atlases have shown that circulating monocytes give rise to *SPP1^+^* macrophages in CRC and CRLM (*32*). Our trajectory analysis extends these observations, suggesting that *SPP1^+^* macrophages are derived from the *THBS1^+^* monocyte population, and with our multiregional approach, we hypothesize that this transition happens at the tumor edge. *SPP1* encodes osteopontin, a protein involved in cell adhesion and migration through interactions with integrins and CD44 (*37*). *SPP1^+^* macrophages have been described as an immunosuppressive population associated with liver metastasis (*10*, *32*), poor survival in CRC (*10*, *16*), and now, based on our data, poor prognosis in CRLM. We derived an *SPP1^+^* macrophage gene signature from our single-cell dataset and showed that it strongly correlates with worse disease-free and overall survival in public CRLM cohorts. Moreover, we found that *SPP1^+^* macrophage abundance positively correlates with exhausted CD8^+^ T cell levels in the same dataset, suggesting that these macrophages contribute to T cell dysfunction within the tumor.

Although we could not directly assess the impact of *SPP1^+^* macrophages on immunotherapy response in CRLM due to limited clinical data, emerging evidence from other cancers supports this connection. For instance, high *SPP1^+^* macrophage infiltration has been linked to poor response to immune checkpoint inhibitors in melanoma, hepatocellular, and esophageal cancers (*38*). *SPP1* neutralization in murine models significantly improved anti–PD-1 responses, reducing breast tumor burden and metastasis (*39*). The genetic knockout of *SPP1* markedly reduced tumor burden and was associated with increased T cell infiltration, activation, and response to anti–PD-1 in CRC (*40*) and anti–programmed death-ligand 1 (PD-L1) in CRLM (*41*). Our cell-cell communication analysis supports this mechanism in the human setting, sugggesting that *SPP1^+^* macrophages in the tumor core interact with exhausted CD8^+^ T cells through immune checkpoint ligand-receptor pairs such as *LGALS9-HAVCR2* (Galectin 9–TIM-3) and *CD86-CTLA4*, which could contribute to immune suppression. In a clinical study of patients with advanced CRC, combined CTLA4 and PD-L1 blockade resulted in only marginal improvement in overall survival (6.6 months versus 4.1 months with supportive care), implying the presence of other immunosuppressive mechanisms (*42*). We identified strong immunosuppressive macrophage–T cell interactions in the tumor core via the *SPP1-CD44* ligand-receptor pair and hypothesize that these interactions contribute to an exhausted CD8^+^ T cell phenotype. In vivo studies support this hypothesis by demonstrating that tumor-reactive CD8^+^ T cells in hepatic metastases preferentially interact with SPP1^+^ macrophages and that SPP1-CD44 signaling promotes T cell exhaustion, which can be reversed by CD44 inhibition (*30*, *31*). Together, the presence of potently immunosuppressive interactions outside of those targeted by current immunotherapy may, at least in part, explain why immune checkpoint blockade alone has shown limited efficacy in these cohorts of patients.

Mechanistically, we propose that the tumor edge serves as a critical immune regulatory hub in CRLM. Here, *THBS1^+^* monocytes engage with *GZMK^+^ CD8^+^* T cells through checkpoint-like pathways (*THBS1-CD47* and *NECTIN2-TIGIT*), promoting T cell dysfunction while reciprocal interactions simultaneously guide monocyte-to-macrophage differentiation via ANXA1-FPR2 signaling. This creates a self-reinforcing circuit whereby monocytes differentiate into *MARCO^+^* and eventually *SPP1^+^* macrophages, which infiltrate the tumor core and further suppress T cell function. These findings position the *THBS1/SPP1* axis as a central orchestrator of immune suppression in CRLM.

From a therapeutic perspective, prior efforts to broadly target monocytes and macrophages, including CSF1R inhibition, have shown limited efficacy as monotherapies in CRC, likely reflecting redundancy and compensatory programs within the myeloid compartment (*43*). These limitations highlight the potential value of targeting specific immunosuppressive subsets over pan-macrophage depletion. In this context, SPP1 represents a mechanistically informed candidate. Although SPP1 is expressed by multiple stromal and immune populations, genetic studies indicate a dominant role for macrophage-derived *SPP1* in immune suppression (*40*, *41*). Notably, in the study by Henriques *et al.* (*41*), *SPP1* knockout reduced CRLMs and synergized with immune checkpoint blockade, while bone marrow transplant restored the immunosuppressive phenotype. Our study with human samples validates these preclinical studies and supports the implication of *SPP1*^+^ macrophages as a key protumorigenic population. Now, the direct pharmacological inhibition of SPP1 remains at the preclinical stage, with neutralizing antibodies (*44*) and macrophage-targeted modulators (*45*) showing efficacy in murine models, and indirect suppression via regulatory pathways is also under investigation.

Together, our study underscores the critical role of spatial organization in shaping the immune microenvironment of human CRLMs. We identify a functional pre-exhaustion zone at the tumor edge and reveal the immunosuppressive contributions of *THBS1^+^* monocytes and *SPP1^+^* macrophages, providing mechanistic insight into immune escape and resistance to immunotherapy. Therapeutically, disrupting the monocyte-to-macrophage trajectory, through inhibition of *THBS1* or *SPP1*, combined with neoadjuvant chemotherapy and immune checkpoint blockade, may offer a promising strategy to reinvigorate antitumor immunity and improve clinical outcomes for patients with CRLM.

## MATERIALS AND METHODS

### Clinical samples

This human study used the PINCER translational platform (*46*) and was approved by the National Research Ethics (NRES) Service Committee North West–Greater Manchester (REC15/NW/0477). All participants provided written informed consent for tissue donation in accordance with institutional protocols. Liver samples were obtained from patients with advanced CRC and liver metastases following completion of neo-adjuvant chemotherapy. Following surgical resection, biopsies were immediately collected from three anatomically distinct regions: the distal NIL (5 to 10 cm away from the tumor core), the tumor edge (0 to 1 cm away from the tumor core), and the tumor core. All diagnoses were confirmed by a Consultant Histopathologist (Clinical information found in table S1).

### Single-cell dissociation of human liver samples

Liver biopsies were dissociated by mechanical and enzymatic disruption with collagenase P (1 mg/ml; Roche) in Hanks’ balanced salt solution at 37°C for 30 min, followed by incubation with 0.05% trypsin (Sigma-Aldrich) at 37°C for 5 min. Cell suspensions were filtered through a 70-μm strainer (Miltenyi) to remove debris, and red blood cells were lysed using RBC Lysis Buffer (BD Pharm Lyse). Immune cells were enriched by density gradient centrifugation with Histopaque-1077 (Sigma-Aldrich) at 400*g* for 30 min at room temperature without brakes. The interface and bottom layer were collected, washed with phosphate-buffered saline (PBS), and resuspended in PBS + 2% bovine serum albumin (BSA) for further use.

### Fluorescence-activated cell sorting

Single-cell suspensions from human tissue were prepared as outlined above, followed by resuspension in MAC buffer (0.5% BSA, 2 mM EDTA, and PBS) and blocking for 10 min on ice with human TrueStain FcX (BioLegend). Cells were then stained with Sytox-blue viability marker (Thermo Fisher Scientific) and fluorophore-conjugated antibodies against CD45 (BioLegend, clone HI30). Fluorescence-activated cell sorting (FACS) was performed on a FACS Aria III (BD Biosciences). Cells were then sorted directly into MAC buffer and placed on ice.

### scRNA-seq sample preparation

Live Sytox*-*CD45^+^ single-cell suspensions from human CRLM were prepared as described above and fixed according to the manufacturer’s protocol. Samples were stored at −80°C for up to 3 months and subsequently submitted for transcriptome library generation using 10X Genomics Chromium platform. Two samples (CB31 and CB46; both tumor edge) failed the partitioning of individual cells in the microfluid chamber and were excluded from further analysis. cDNA libraries were sequenced using the Illumina NovaSeq S1 instrument.

### Alignment and quantification of counts

Raw FASTQ sequencing data were analyzed and aligned to the GRCh38 human reference genome with CellRanger (3.0.2). The unique molecular identifier (UMI) counts were summarized (UMI) for each gene of each cell. The raw UMI count matrices were then converted into a Seurat v5 object (*47*) in R (4.3.1). Cells with a low number of genes <200 (indicating low quality) or high numbers of genes >6000 (suggesting doublets) were removed from the dataset. Last, cells with more than 15% mitochondrially encoded genes were filtered out from analysis.

### Data integration, normalization, and dimensionality reduction

Data were scaled with the top 2000 most variable genes by using the FindVariableFeatures function. These variable genes were used for principal components analysis, and the FindNeighbors function was used to obtain a nearest neighbor map between cells across the number of PCs determined by the ElbowPlot function. To eliminate batch effects between samples, we performed data integration with the Harmony R package (*48*). The FindClusters function was used to obtain cell clusters with cluster numbers being determined by varying resolution settings. All functions described were from Seurat v5 unless otherwise stated.

### Differential expression

To annotate and identify marker genes for clusters, DEGs were identified by a nonparametric Wilcoxon rank-sum test implemented in the FindAllMarkers function. Genes were filtered for a log fold change of 0.25 and expressed in at least 25% of cluster cells.

### Gene signature enrichment analysis

To infer MoM functions, DEGs between each cluster were determined by the FindMarkers function in Seurat, with the following parameters: only.pos = T, logfc.threshold = 0.1, and min.pct = 0.1 (*47*). Significant DEGs were then ranked according to their log fold change. The ranked genes were used in the gseGO function implemented by clusterProfiler (4.8.3). The maximum gene set size was 500, the “BP” ontology was used, and *P* values were adjusted using the Benjamini-Hochberg method (*49*).

### Tissue distribution of clusters

Cluster enrichment across the three locations was quantified by comparing the observed (_O_) and expected (_E_) cell frequencies in each cluster according to the formula described previously (*R*_O/E_) (*50*). The expected frequencies for each combination of cell clusters and locations were calculated with a Chi-square test. *R*_O/E_ > 1 indicated enrichment.

### Pseudotime analysis

Linear pseudotime ordering was performed for the CD8 T cell clusters using the Bayesian latent variable statistical framework Ouija (*51*), with 10,000 iterations. To expedite the calculations, a random subsample of 800 cells per patient from this T cell subset was used as input. A set of 23 selected marker genes (table S9) was utilized for pseudotime learning, and individual cells were ranked on the basis of their assigned pseudotime values for subsequent analysis. The mean pseudotime for each sample and clusters was calculated as the average pseudotime rank of all CD8^+^ T cells from the respective sample/cluster included in the Ouija analysis.

### Trajectory inference

To investigate the trajectory of MoM differentiation, pseudotime analysis was conducted on a subset of monocytes and MoMs using Monocle3 (v1.3.4) (*52*). On the basis of previous analyses, we selected Mono_c0_*THBS1* as the root for the trajectory. We partitioned the clusters using the cluster_cells function with a resolution of 1e-3 and constructed a principal graph using the learn_graph function. The order_cells function was then used to assign cells a pseudotime value, which was then mapped onto the Uniform Manifold Approximation and Projection for further visualization and interpretation.

### Survival analysis of microarray gene expression data

Publicly available microarray data were downloaded from the Gene Expression Omnibus under the accession GSE159216 (*20*). A 10-gene *SPP1*^+^ macrophage signature was derived from the most up-regulated genes within this cluster from our in-house scRNA-seq dataset. An *SPP1*-associated gene programme score was computed using ssGSEA (GSVA v2.2.0) (*53*). Patients were dichotomized into high and low *SPP1* signature groups using the surv_cutpoint function (minimum 20% per group). Kaplan–Meier survival curves were generated with survfit and visualized using ggsurvplot.

### Cell-type proportion deconvolution of microarray data

We uploaded a reference signature matrix from our scRNA-seq dataset to CIBERSORTx (*54*) to estimate cell type proportions within the GSE159216 dataset. Quartile normalization was disabled, S-mode batch correction was applied, permuations were set to 500, and other parameters were used with default settings. Spearman’s correlation analysis was used to investigate the relationship between proportions of cell types of interest.

### Cell interaction analysis

Subclustered CD8^+^ T cells, CD4^+^ T cells, and myeloid cell datasets were merged without integration with the merge function in Seurat v5. The merged object was then subsetted to generate two Seurat objects with cells from the tumor core and tumor edge. All functions described herein are from the CellChat package (v2.1.2) (*55*). Each object was processed individually with default settings to create two CellChat objects with the createCellChat function. The subsetDB function was run to include ligand-receptor pairs associated with “Secreted Signaling,” “ECM-Receptor,” and “Cell-Cell Contact,” Following this, each object was merged with the mergeCellChat function. Differential interaction strengths were calculated with the netVisual_diffInteraction function with the measure = “weight” parameter set. To identify populations of cells with significant changes in outgoing or incoming L-R signaling between the two locations, the netAnalysis_computeCentrality function with a threshold of 1 × 10^−4^ was run on the merged CellChat object. netVisual_bubble was run to identify signaling probabilities between specific clusters of cells.

### Mass cytometry sample preparation

Single-cell suspensions were prepared and washed with Maxpar PBS (Standard Biotools). The cell pellet was resuspended in Maxpar PBS and stained with 1:1000 dilution of Cell-ID 103-Rhodium viability marker (Standard Biotools) for 15 min at room temperature. After staining, cells were washed in Maxpar cell staining buffer (Standard Biotools) and fixed with 1.6% paraformaldehyde (Thermo Fisher Scientific) for 10 min on ice. Cells were then centrifuged at 800*g* for 5 min and stored at −80°C.

Upon thawing on ice, the cells were washed in Maxpar PBS, permeabilized on ice with Maxpar Barcode Perm Buffer (Standard Biotools) for 10 min, and barcoded using 20-Plex Pd Barcoding (Standard Biotools) for 10 min. After two washes in PBS, samples were pooled together. For surface staining, cells were incubated with a metal-conjugated antibody cocktail (table S10) for 40 min at 4°C. Following staining, cells were washed twice with cell staining buffer and stained overnight at 4°C with 125 μM Intercalator-191Ir (Standard Biotools) diluted 1:2000 in cell staining buffer.

### Sample acquisition and mass cytometry data analysis

Cells were washed in Maxpar cell staining buffer and resuspended in Cell Acquisition Solution (Standard Biotools) supplemented with 0.1X EQTM Four Element Calibration Beads (Standard Biotools). Acquisition was performed on a Helios CyTOF system (Standard Biotools) at a rate of <500 events per second. FCS files were normalized using EQ beads standards to ensure consistent calibration across samples. Data analysis was performed using FlowJo, with manual gating to exclude debris, identify single cells (191Ir^+^) and differentiate live and dead cells (103Rh^+^). viSNE analysis was applied to the high-dimensional data for visualization. Automated analysis was then performed using the FlowSOM plugin in FlowJo, enabling the identification and automatic annotation of distinct cellular subsets based on their marker profiles. Markers used for clustering were as follows: CD11b, CD8a, CD172ab, CD141, CD3, CD14, CD16, CD19, CD4, CD56, CD11c, CD15, HLADR, BCDA2, and PD-1. CD4^+^ T cells were subclustered using CD56, CD16, IL7-Ra, PD-1, LAG3, HLADR, TIGIT, CD25, CD45RO, and CD69. CD8^+^ T cells were subclustered using CD56, PD-1, TIGIT, CD25, CD16, LAG3, IL7-Ra, CD45RO, and CD69.

### Hematoxylin and eosin staining

FFPE human liver samples were deparaffinized in xylene and rehydrated through a graded ethanol series. Sections were stained with hematoxylin for 5 min, rinsed in water, and counterstained with eosin for 1 min. After a final wash, slides were dehydrated through ascending ethanol concentrations, cleared in xylene, and mounted.

### Immunostaining

Deparaffinization and antigen retrieval was performed on tissue sections with the PT-Link System (Dako), followed by blocking with 10% normal donkey serum. Sections were then incubated with primary antibodies at 4°C overnight followed by incubation with fluorophore-conjugated secondary antibodies (table S11) and nuclear dye 4′,6-diamidino-2-phenylindole (Thermo Fisher Scientific) at room temperature for 2 hours. Stained sections were then mounted with Fluorescent Mounting Medium (Dako). Images were acquired with an Axio Observe Z1 microscope with the Apotome.2 (Zeiss). Data were analyzed on Zen Blue (3.1.0, Zeiss). From each patient, five fields of view per section were quantified and averaged for each data point.

### Spatial analysis

Spatial analysis was conducted using QuPath and Python, following protocols outlined by Akoya Biosciences (*56*). QuPath was used for image visualization and cell segmentation, while Python facilitated data processing, phenotyping, and spatial metrics computation.

QuPath (v0.4.3) was used for project setup and image analysis (*57*). The StarDist extension was integrated into QuPath to enable precise cell segmentation (*58*). Following segmentation, data were exported from QuPath and imported into Python using the Scanpy library, creating an AnnData object that encapsulated expression data, spatial coordinates, and cell metadata. Automated phenotyping was performed by rescaling data using a two-class Gaussian mixture model, converting raw intensities to probabilities. Cells were classified based on marker positivity (CD8^+^ as CD8 T cells, IBA1^+^ as macrophages, CD14^+^IBA1^−^ as monocytes, and CD14^+^IBA1^+^ as MoMs).

As previously reported (*21*), to assess the spatial relationships among MoMs, CD8^+^ T cells, and monocytes, a SpatialScore was computed on the basis of the relative proximity of CD8^+^ T cells to MoMs and monocytes. The SpatialScore was defined as

$$\text{Spatial Score}=\frac{\mathrm{dx}}{dy}$$

where dx represents the distance between a CD8^+^ T cell and the nearest MoM and dy represents the distance between a CD8^+^ T cell and the nearest monocyte. A lower spatial score indicates that CD8^+^ T cells are closer to CD14^+^IBA1^+^ MoMs than CD14^+^IBA1^−^ monocytes, while a higher spatial score suggests that CD8^+^ T cells localize more closely with CD14^+^IBA1^−^ monocytes than they do with CD14^+^IBA1^+^ MoMs.

### Statistical analysis

Statistical analyses were performed using GraphPad Prism v8 or Python. One-way analysis of variance (ANOVA) with Tukey’s post hoc correction was used for Fig. 2H and fig. S5E, while a two-sided Wilcoxon’s rank-sum test was used for Fig. 4C. A *P* value < 0.05 was considered statistically significant. Statistical significance is indicated in the figures as follows: *P < 0.05, **P < 0.01, ***P < 0.001, and ****P < 0.0001; ns denotes not significant.

### Patient and public involvement statement

Patient input was sought to ensure that the research aims and procedures were communicated clearly, and their feedback was instrumental in shaping how study participation was presented to prospective tissue sample donors. While patients were not directly involved in the design, conduct, or reporting of the study, they contributed to the development of the consent process, including the wording of consent materials. This study used anonymized tissue samples and clinical data from patients with CRLMs who provided informed consent for research use.
