# Supplementary material for: Multiregional profiling reveals THBS1-SPP1 monocyte-macrophage axis drives immunosuppression and outcome in colorectal liver metastases
Source: Sci Adv. 2026 May 22;12(21):eaed1296. doi: 10.1126/sciadv.aed1296 (PMC13196778; doi:10.1126/sciadv.aed1296)
Supplement: Supplementary file 1 — Figs. S1 to S8 Legends for tables S1 to S11 [file sciadv.aed1296_sm.pdf]

Science Advances
AAAS

Supplementary Materials for

Multiregional profiling reveals *THBS1-SPP1* monocyte-macrophage axis drives immunosuppression and outcome in colorectal liver metastases

Gaia Bellomo *et al.*

Corresponding author: Michael C. Schmid, mschmid@liverpool.ac.uk; Ainhoa Mielgo, amielgo@liverpool.ac.uk

Sci. Adv. **12**, eaed1296 (2026)
DOI: 10.1126/sciadv.aed1296

The PDF file includes:

Figs. S1 to S8
Legends for tables S1 to S11

Other Supplementary Material for this manuscript includes the following:

Tables S1 to S11

Supplementary Figures 1-8

Suppl. Fig. 1: Whole-slide H&E scan of the patient's samples

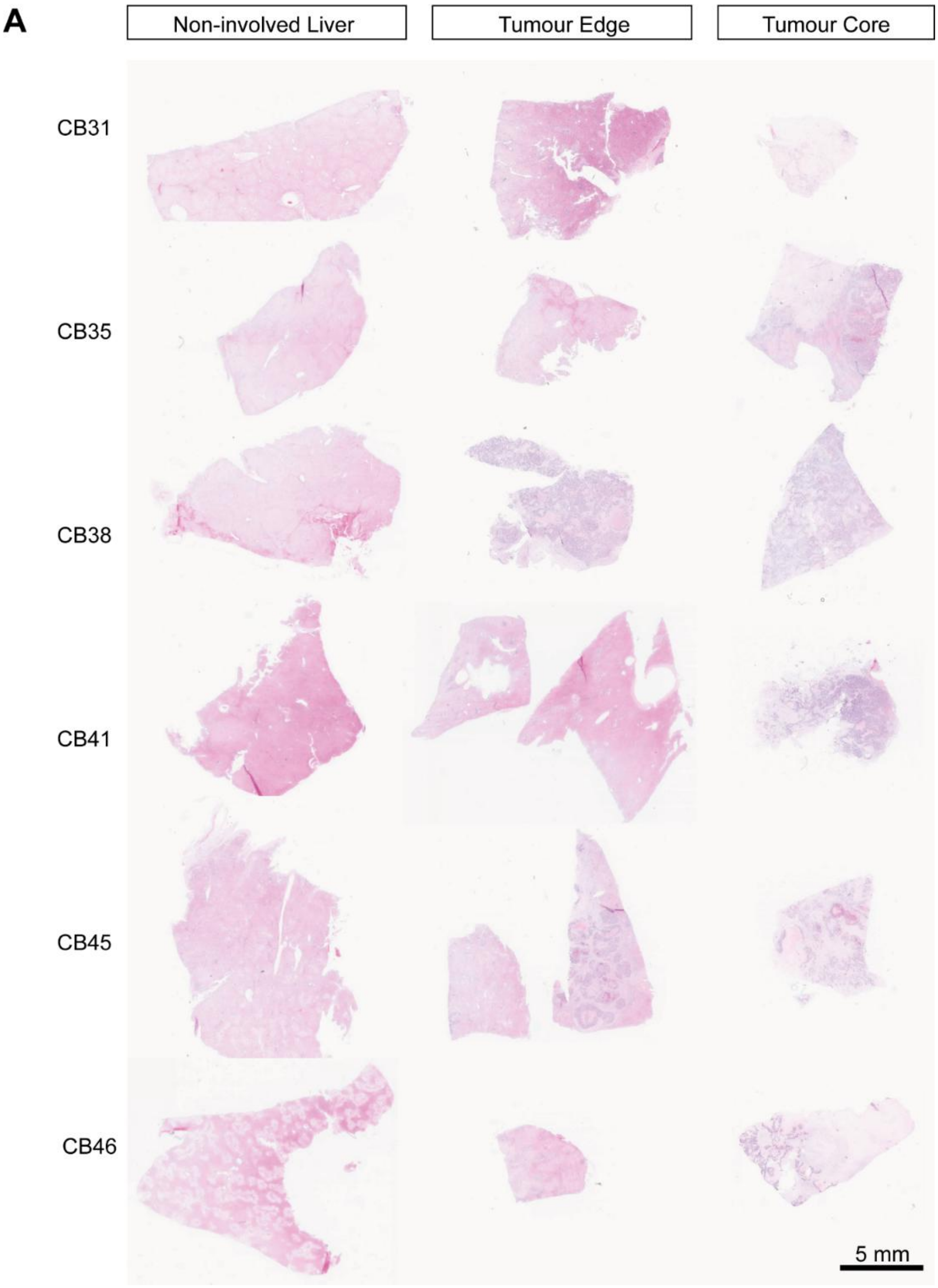

Suppl. Fig. 1: Whole-slide H&E scan of the patient's samples. (A) H&E-stained tissue from the patient included in this study, showing the overall histological architecture of NIL, tumour edge and core. Scale bar = 5 mm.

Suppl. Fig. 2: scRNA-seq reveals distinct immune populations across CRLM regions

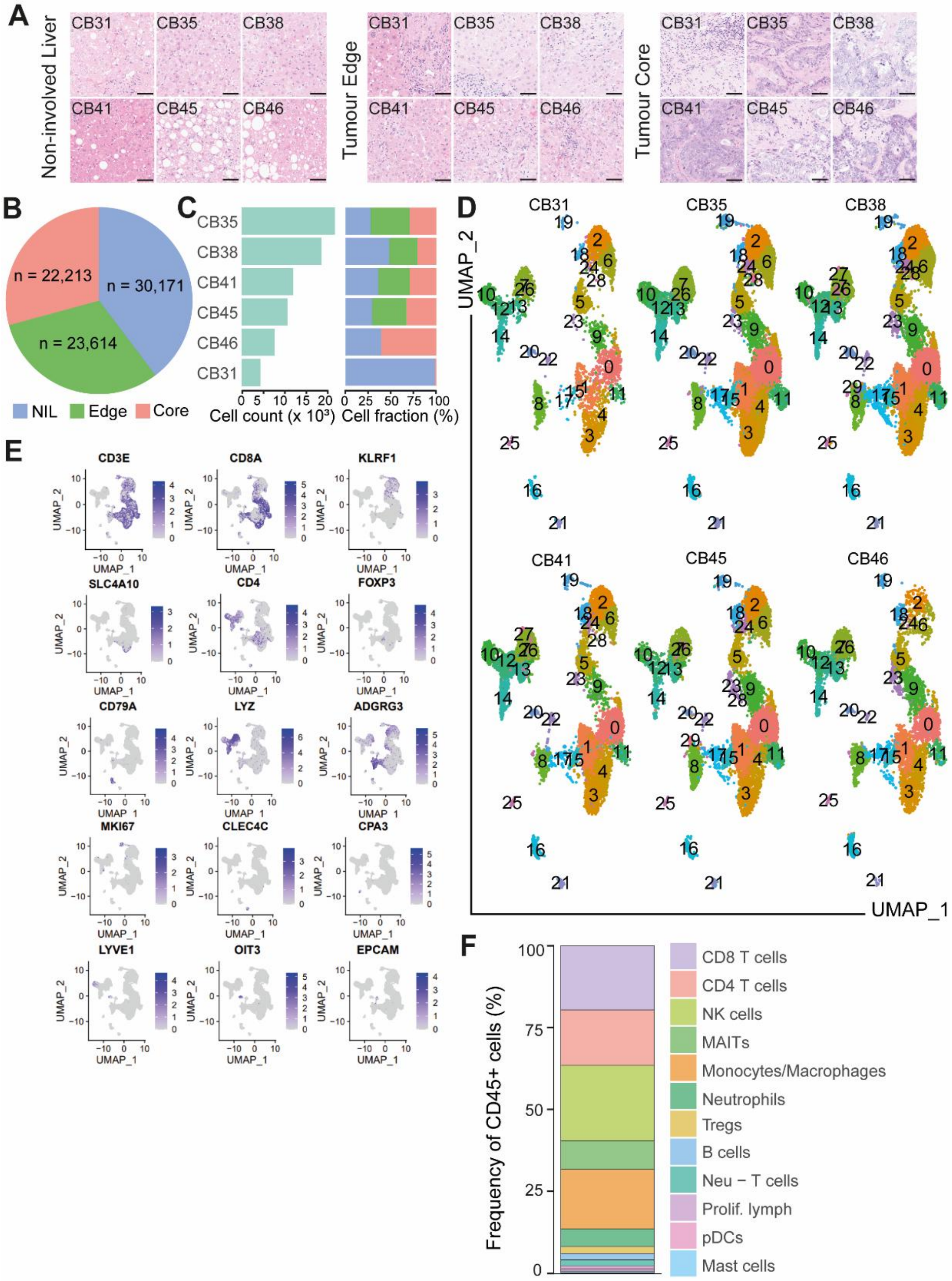

Suppl. Fig. 2: scRNA-seq complemented by mass cytometry reveals distinct immune populations across CRLM regions. (A) Selected ROI from H&E-stained tissue from the patient included in this study. Scale bar = 50µm. **(B)** Pie chart showing the proportion of cells derived from the NIL, tumour edge and core. **(C)** Histogram showing the cell count per patient (left). Stacked bar plot illustrating the proportion of cells contributing to each region per patient (right). **(D)** UMAP embedding from the joint analysis shown separately for each patient, demonstrating consistent representation of clusters across patients. **(E)** Feature plots showing lineage marker genes. **(F)** Stacked bar plot showing the overall proportion of major immune cell populations in CRLM by scRNA-seq.

Suppl. Fig. 3: Mass cytometry reveals distinct immune populations across CRLM regions

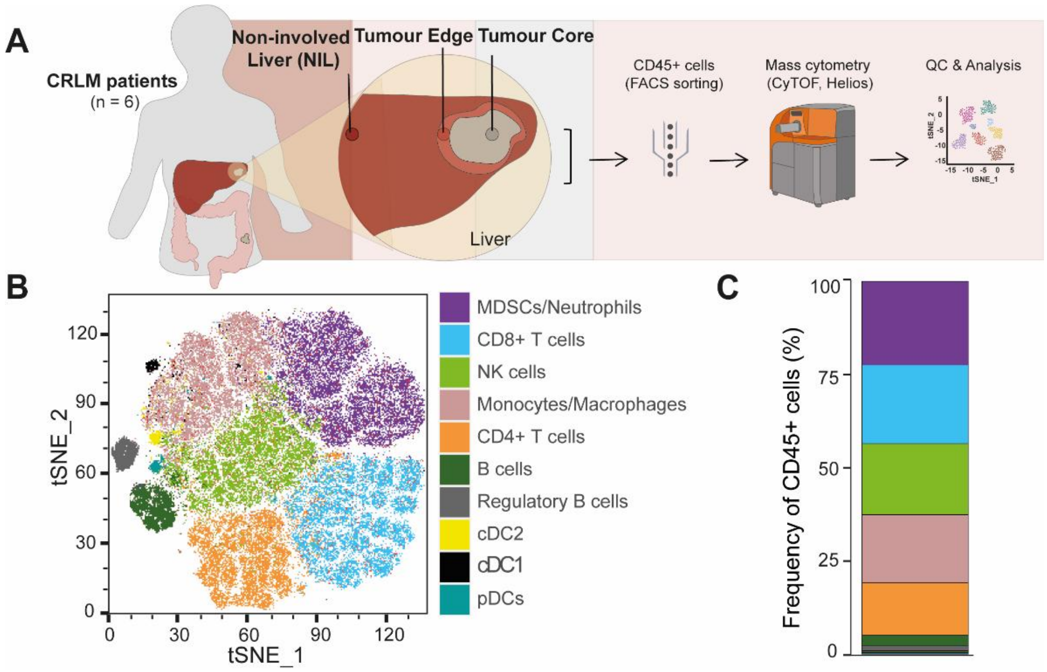

Suppl. Fig. 3: Mass cytometry reveals distinct immune populations across CRLM regions. (A) Schematic depicting the sampling of CRLM from the NIL, tumour edge, and tumour core, followed by tissue processing, $CD45^{+}$ immune cell sorting, mass cytometry, and data analysis. **(B)** t-SNE plot depicting the distribution of major immune cell populations in CRLM as determined by mass cytometry. **(C)** Stacked bar plot showing the overall proportion of major immune cell populations in CRLM identified by mass cytometry.

Suppl. Fig. 4: CD4$^+$ and CD8$^+$ T cell cluster distribution in CRLM

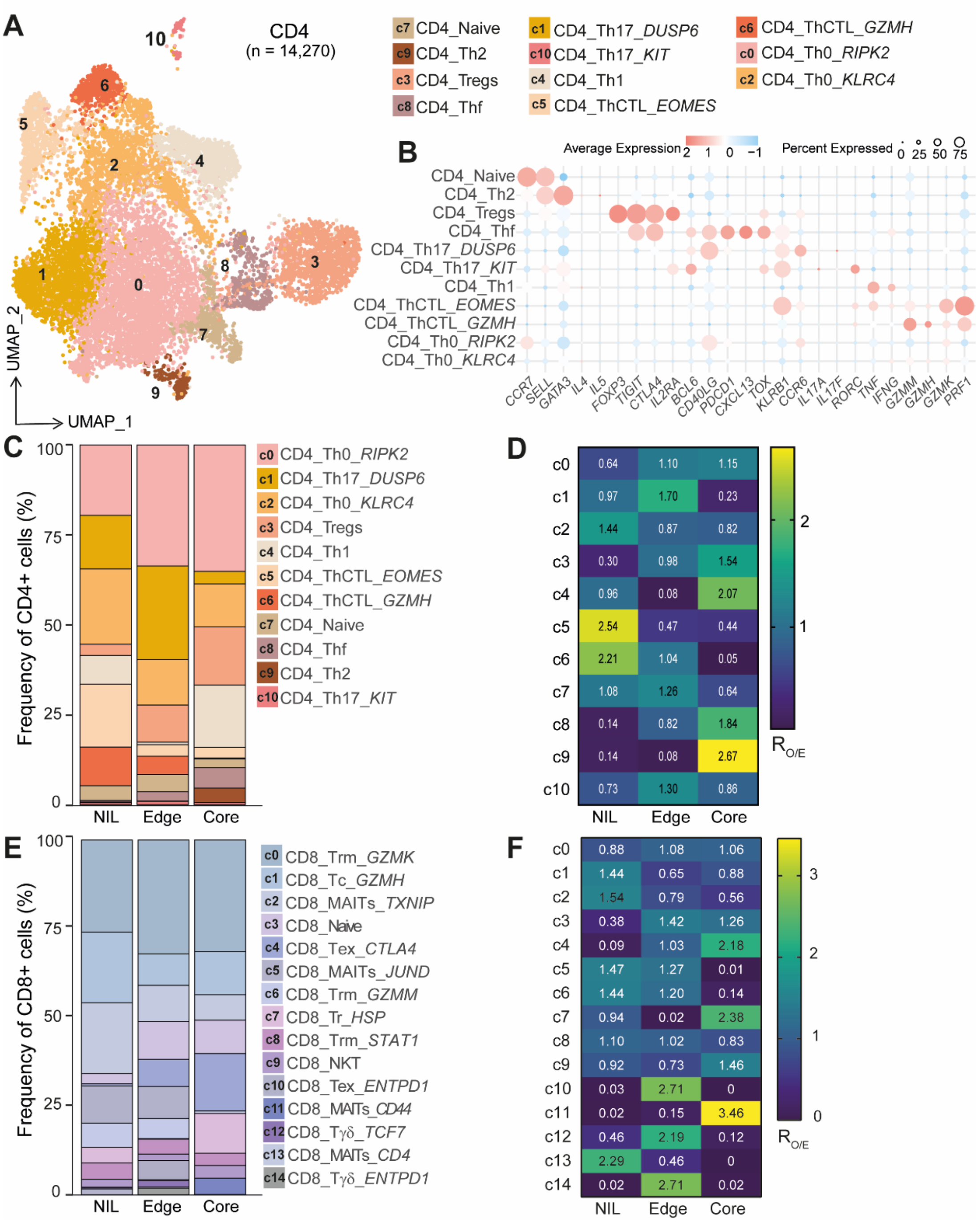

Suppl. Fig. 4: CD4$^+$ and CD8$^+$ T cell cluster distribution in CRLM. (A) UMAP plot identifying eleven CD4$^+$ T cell clusters. **(B)** Dot plot displaying gene markers used for cluster annotation. **(C)** Stacked bar plots showing CD4$^+$ T cell clusters distribution in NIL, tumour edge and core. **(D)** Heat map of CD4$^+$ T cell clusters tissue prevalence estimated by $R_{O/E}$. **(E)** Stacked bar plots showing CD8$^+$ T cell clusters distribution in NIL, tumour edge and core. **(F)** Heat map of CD8$^+$ T cell clusters showing cluster enrichment per location estimated by $R_{O/E}$.

Suppl. Fig. 5: Progressive CD8⁺ T cell exhaustion from NIL to tumour core

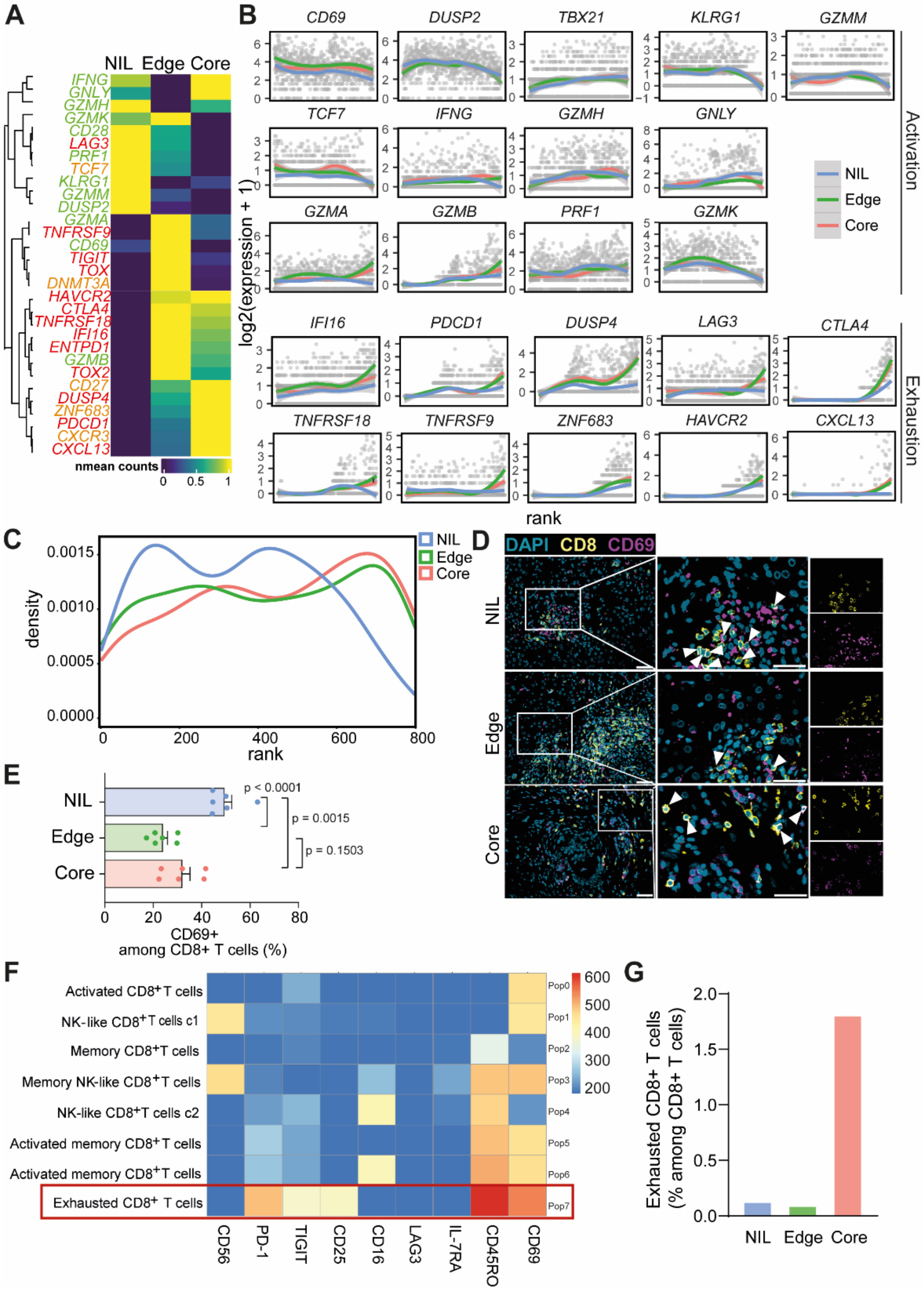

Suppl. Fig. 5: Progressive $CD8^+$ T cell exhaustion from NIL to tumour core. A) Heatmap showing normalized average expression of selected genes associated with T cell activation and/or exhaustion for each for each CRLM zone. **(B)** Single-cell expression of the indicated activation and exhaustion genes ordered by pseudotime. The analysis was done on scRNA-seq data from $CD8^+$ T cells in all samples. The lines correspond to LOESS curve for each zone. **(C)** Density plot depicting pseudotime-ordered $CD8^+$ T cells based on scRNA-seq data. Lines in the plot are coloured according to CRLM zone. **(D)** Representative immunofluorescent images and **(E)** quantification of active $CD8^+$ T cells ($CD8^+CD69^+$) in NIL, tumour edge and tumour core. Scale bar = 50µm. Error bars, mean ± s.e.m. P values, one-way ANOVA with Tukey's post-hoc test. **(F)** Heat map displaying T cell surface marker expression from mass cytometry analysis used for clustering with FlowSOM. **(G)** Histogram showing exhausted $CD8^+$ T cells frequency among $CD8^+$ T cells in the NIL, tumour edge, and tumour core, derived from mass cytometry analysis.

Suppl. Fig. 6: Myeloid cell clusters distribution in CRLM

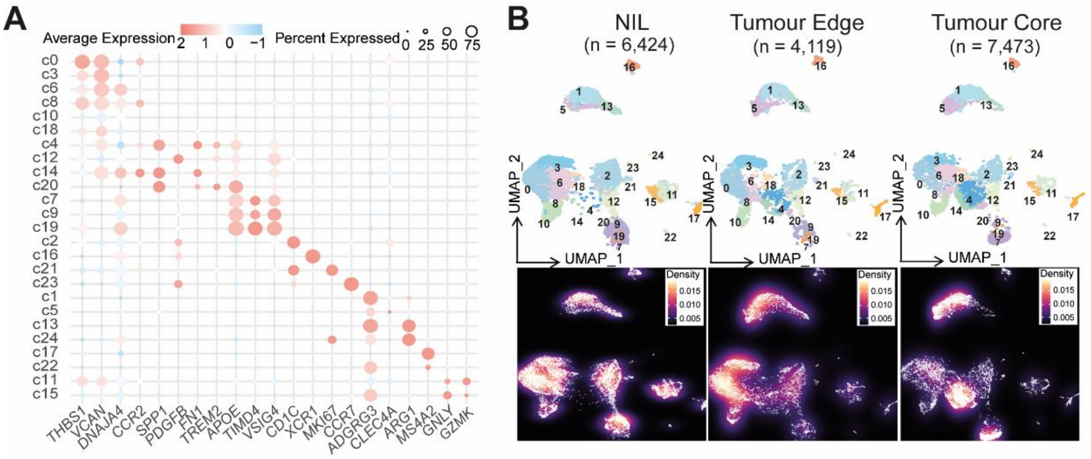

Suppl. Fig. 6: Myeloid cell clusters distribution in CRLM. (A) Dot plot displaying gene markers used for myeloid clusters annotation. **(B)** UMAP view of myeloid clusters (top) and cell density (bottom) displaying myeloid cell distribution across NIL, tumour edge and core.

Suppl. Fig. 7: Immunosuppressive MoM clusters localize to the tumour core and display distinct gene programs

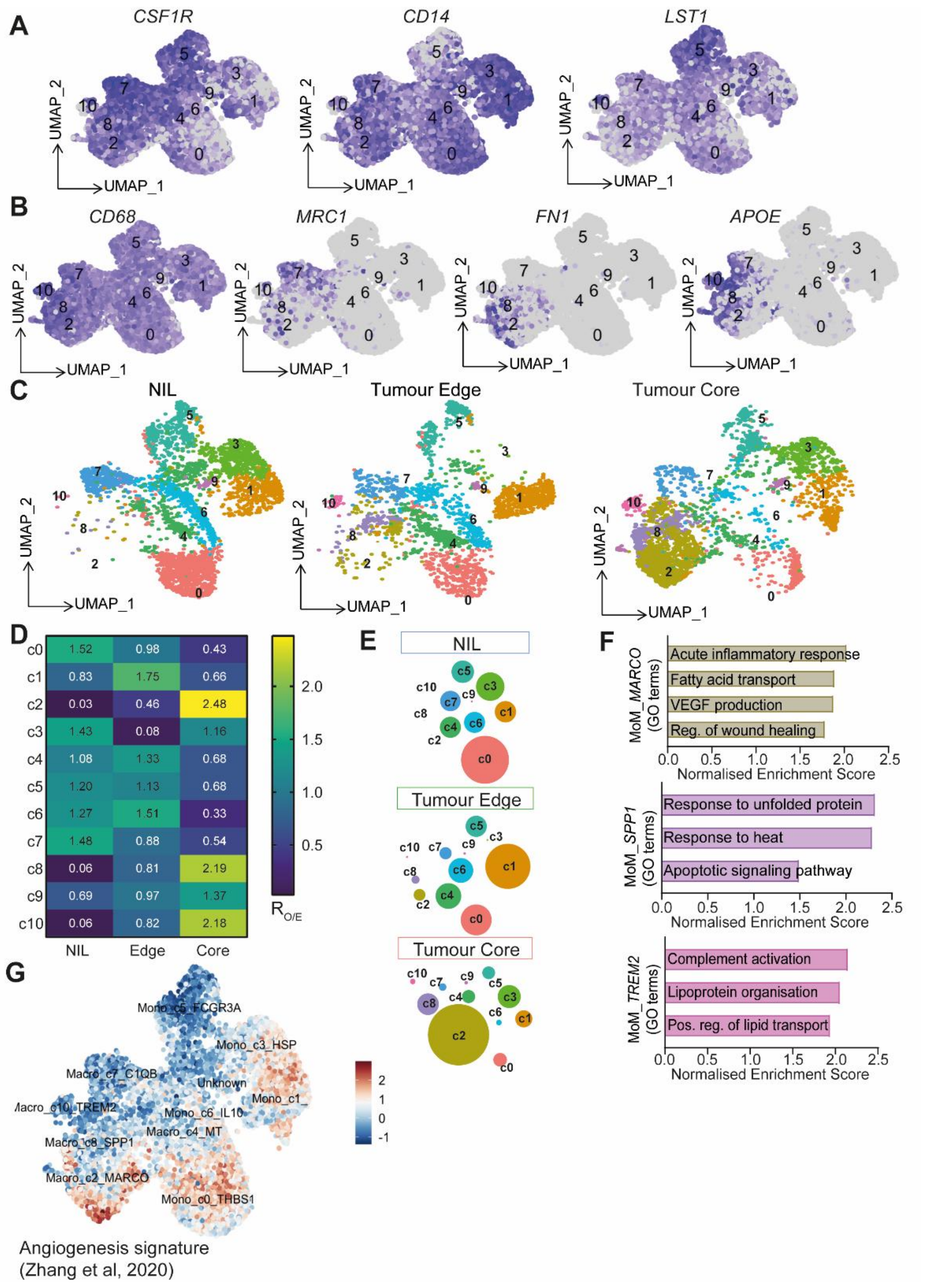

Suppl. Fig. 7: Immunosuppressive MoM clusters localize to the tumour core and display distinct gene programs. (A) Feature plots displaying expression of monocyte-associated genes. **(B)** Feature plots displaying expression of macrophage-associated genes. **(C)** UMAP showing distribution of eleven Monocyte/MoM clusters in NIL, tumour edge and core. **(D)** Heat map of Mono and MoM clusters showing tissue prevalence estimated by $R_{O/E}$. **(E)** Diagram showing distribution of Mono and MoM clusters across NIL, tumor edge and core. **(F)** Enriched Gene Ontology biological processes (BP) in immunosuppressive MoM clusters derived from gene set enrichment analyses using the *gseGO* function in clusterProfiler. **(G)** UMAP plot showing single-cell distribution coloured by the angiogenesis signature (30) scores computed using the *AddModuleScore* function in Seurat v5.

Suppl. Fig. 8: Cell-cell interaction analysis in tumour edge and tumour core

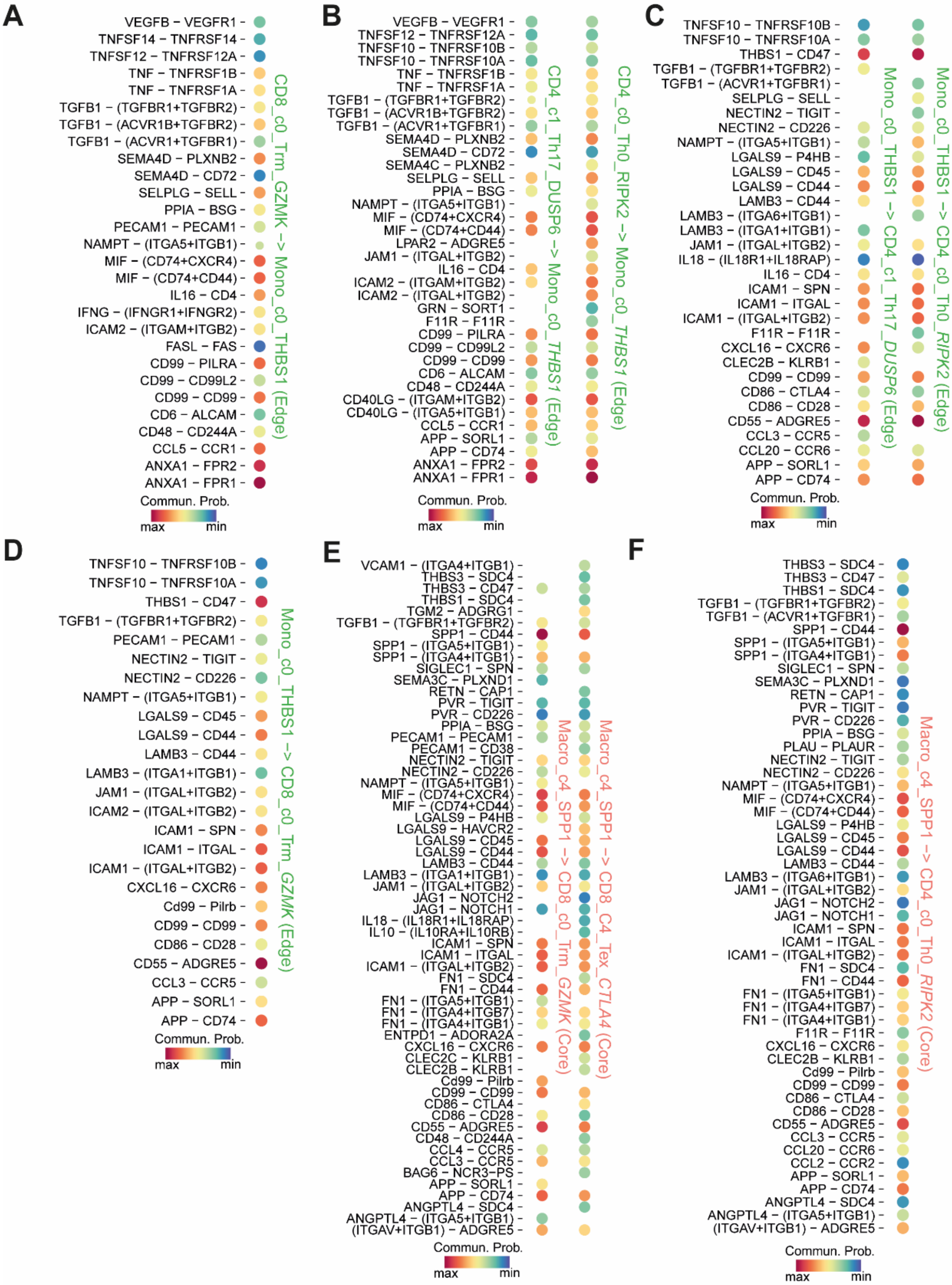

Suppl. Fig. 8: Cell-cell interaction analysis in tumour edge and tumour core. (A) Dot plot depicting LR interactions from CD8^{+} T cell clusters to THBS1^{+} monocytes, enriched in the tumour edge. **(B)** Dot plot depicting LR interactions from CD4^{+} T cell clusters to THBS1+ monocytes, enriched in the tumour edge. **(C)** Dot plot depicting LR interactions from THBS1^{+} monocytes to CD4^{+} T cell clusters, enriched in the tumour edge. **(D)** Dot plot depicting LR interactions from THBS1^{+} monocytes to CD8_c0_Trm_*GZMK*, enriched in the tumour edge **(E)** Dot plot depicting LR interactions from SPP1^{+} macrophages to CD8^{+} T cell clusters, enriched in the tumour core. **(F)** Dot plot depicting LR interactions from SPP1^{+} macrophages to CD4_c0_Th0_*RIPK2*, enriched in the tumour core. In **(A-F)**, colour intensity represents the probability of communication.

Supplementary Table 1 - Clinical annotations of patients included in the study cohort.

Supplementary Table 2 - DEG list for all cells.

Supplementary Table 3 - DEG list for CD4+ cells.

Supplementary Table 4 - DEG list for CD8+ cells.

Supplementary Table 5 - DEG list for myeloid cells.

Supplementary Table 6 - DEG list for monoyctes/macrophages.

Supplementary Table 7 - Genes comprising the SPP1 signature.

Supplementary Table 8 - Quantitative scores for outgoing and incoming cell-cell interactions inferred by CellChat.

Supplementary Table 9 - Genes used for pseudotime inference using Ouija.

Supplementary Table 10 - Antibody panel used for mass cytometry analysis.

Supplementary Table 11 - Primary and secondary antibodies used for immunofluorescence staining.
